# Introduction: MHC/KIR and governance of specificity

**DOI:** 10.1007/s00251-017-0986-6

**Published:** 2017-07-10

**Authors:** Adrian Kelly, John Trowsdale

**Affiliations:** Department of Pathology, University of Cambridge, Cambridge, CB21QP UK

**Keywords:** MHC, HLA, KIR, Antigen processing, Antigen presentation

## Abstract

The MHC controls specificity, to ensure that appropriate immune responses are mounted to invading pathogens whilst maintaining tolerance to the host. It encodes molecules that act as sentinels, providing a snapshot of the health of the interior and exterior of the cell for immune surveillance. To maintain the ability to respond appropriately to any disease requires a delicate balance of expression and function, and many subtleties of the system have been described at the gene, individual and population level. The main players are the highly polymorphic classical MHC class I and class II molecules, as well as some non-classical loci of both types. Transporter associated with antigen processing (TAP) peptide transporters, proteasome components and Tapasin, encoded within the MHC, are also involved in selection of peptide for presentation. The plethora of mechanisms microorganisms use to subvert immune recognition, through blocking these antigen processing and presentation pathways, attests to the importance of HLA in resistance to infection. There is continued interest in MHC genetics in its own right, as well as in relation to KIR, to transplantation, infection, autoimmunity and reproduction. Also of topical interest, cancer immunotherapy through checkpoint inhibition depends on highly specific recognition of cancer peptide antigen and continued expression of HLA molecules. Here, we briefly introduce some background to the MHC/KIR axis in man. This special issue of *immunogenetics* expands on these topics, in humans and other model species.

## Introduction

HLA is the earliest and most widespread example of personalised medicine, in relation to transplantation biology. It is also the region of the genome associated genetically with more diseases than any other, including infectious conditions, but particularly autoimmunity. Resistance to disease is thought to drive the extreme polymorphism of HLA, but other mechanisms have been proposed. In the last two decades, attention has turned to unexpected roles for MHC molecules such as in modelling of synapses (Shatz 2009) and, as discussed in this volume by Colucci and by Persson et al., pregnancy. In support of the former, many different microbial species, particularly viruses and bacteria, elaborate proteins that block antigen processing and presentation. The human MHC encompasses over 200 genes, at least 50 of which have functions in immune defence. They include the following: three classical and three non-classical class I loci, plus several pseudogenes; four classical and two non-classical class II loci, each comprising two genes (A and B chains); four complement components; two immune regulators; 10 genes involved in inflammation or activation of NK cells; three genes involved in stress responses; six genes to do with leukocyte maturation; five genes encoding antigen processing elements; and two other IgSF molecules. Transplantation is discussed in this volume in relation to allorecognition and viruses by Frans Claas.

### Class I processing and presentation

MHC class I molecules have evolved mainly to present peptides from pathogens that replicate within host cells, in particular viruses. The presence of three classical class I isotypes HLA-A, HLA-B and HLA-C, with different peptide-binding characteristics, ensures that pathogens with different protein coding capacity remain visible to the immune system. Alleles of all three isotypes utilise a common pathway for peptide loading. In brief, protein from the pathogen is first degraded in the cytosol, and the resulting peptides are then translocated into the endoplasmic reticulum. Here, they are loaded onto class I molecules before transit to the cell surface for presentation to cytotoxic CD8 T cells.

The proteasome, a cytosolic protease, is largely responsible for generating the peptides that eventually bind class I molecules. It comes in various forms. In many cell types, MHC peptide presentation is linked to constitutive protein turnover and is undertaken by a proteasome that utilises three catalytic subunits termed β1, β2 and β5. In professional antigen presenting cells, or in the presence of interferon, these subunits can be exchanged for βi, β2i and β5i to produce an immunoproteasome, which tailors peptides for MHC binding (Gaczynska et al. 1993). The immunoproteasome has altered specificity and preferentially generates peptides with C-terminal hydrophobic or basic residues. These amino acids are used by the majority of class I alleles to anchor the C-terminus of the peptide into the MHC molecule (Gaczynska et al. 1993; Young et al. 1995). The subunit composition of the immunoproteasome can be further modified by substitution of β5i with β5t to generate the thymoproteasome. The β5t subunit confers reduced chymotrypsin-like activity thereby influencing the generation of peptides with hydrophobic C-terminal residues, amino acids favoured by 90% of MHC alleles (Takahama et al. 2008). Expression of the thymoproteasome by thymic cortical epithelial cells may assist thymic education by generating fewer high affinity peptides and in doing so prevent excessive loss of T cells through negative selection (Takahama et al. 2008; Tomaru et al. 2009).

Once generated by the proteasome, peptides must be rapidly transported into the endoplasmic reticulum to escape the activity of cytosolic aminopeptidases, which would otherwise lead to their rapid degradation (Reits et al. 2003). This is mediated by the transporter associated with antigen processing (TAP), a heterodimer of TAP1 and TAP2 that is encoded in the MHC (Kelly et al. 1992). Human TAP preferentially transports peptides that are 8–12 aa in length (Androlewicz and Cresswell 1996). In contrast to other species, it is promiscuous in the diversity of peptides translocated, and so for humans, the class I molecule itself largely determines which peptides are ultimately presented to the immune system (Androlewicz and Cresswell 1996). Although there is limited flexibility in the length of peptide that MHC I molecules can accommodate, those that are too long to bind particular alleles can be trimmed at the amino-terminus by ER resident peptidases ERAP1 and ERAP2 (Chen et al. 2016). A related enzyme, insulin-regulated aminopeptidase (IRAP), performs a similar function in endosomal compartments and is important in cross-presentation (Saveanu et al. 2009). In addition to its transport function, TAP forms the central pillar of the peptide-loading complex, an assembly of proteins that ensure efficient loading of peptide onto MHC class I (Blum et al. 2013; Cresswell et al. 1999; Neefjes et al. 2011). After initial association with calnexin, empty MHC I/β2 enters a complex comprising TAP, two general folding chaperones, ERp57 and calreticulin, and tapasin, a dedicated class I chaperone (Cresswell et al. 1999). Tapasin functions to bridge class I molecules awaiting peptide with TAP and is essential for the efficient optimization of class I peptide selection (Williams et al. 2002). A molecular tug-of-war between peptide and tapasin is the basis of the selection process (Fisette et al. 2016). Tapasin exerts force upon the peptide-binding domain resulting in opening of the grove and release of low affinity peptide. High affinity peptide binds strongly to the MHC molecule and is able to overcome the force exerted by tapasin resulting in closure of the groove and release of tapasin (Fisette et al. 2016). Interestingly, some class I alleles optimise peptide selection in the complete absence of tapasin, possibly as a mechanism to counter pathogen subversion (Williams et al. 2002). For these tapasin-independent alleles, peptide optimization is poorly enhanced when tapasin is present (Williams et al. 2002). More recently TAPBPR, a second tapasin-related MHC I chaperone, has been shown to enhance peptide optimisation and also reduce the diversity of peptides presented by MHC class I (Hermann et al. 2015). How these two chaperones cooperate is yet to be determined, but ultimately class I with its peptide cargo arrives at the cell surface for presentation to T cells. Recently, attention has focused on the association of certain HLA-B and HLA-C alleles and resistance to disease (Carrington et al. 1999). Whilst this might be predictable given their role in immune surveillance, an unexpected correlation with the level of cell surface expression of HLA-C and prevalence of cytotoxic T cell responses to HIV was observed (Apps et al. 2013). Increased surface expression may correlate with a more focused peptide repertoire and may be a more general feature of MHC biology than previously appreciated (Chappell et al. 2015).

The MHC also encodes three non-classical molecules HLA-E, HLA-F and HLA-G that show limited polymorphism, covered here by Persson et al. The best defined of these is HLA-E which interacts with inhibitory receptors present on the surface of NK cells to prevent killing but may also present peptides from microorganisms to T cells (Braud et al. 1998; van Meijgaarden et al. 2015). It is limited in the diversity of peptides it can bind, preferentially accommodating peptides that originate from signal sequences derived from classical HLA-A, HLA-B and HLA-C (Miller et al. 2003). Interference in the expression of classical class I molecules, such as occurs during viral infection, may be detected by ‘missing-self’, or lack of surface HLA-E expression. The functions of HLA-F and HLA-G are less well-characterised. HLA-G expression was first reported in placental trophoblast cells and a role in acceptance of the foetus ‘allograph’ proposed. Here, HLA-G may influence the function of NK cells directly by interaction with receptors on NK cells, monocytes in the form of an HLA-G dimer (Clements et al. 2005), or indirectly by supporting the expression of surface HLA-E. Caution has been suggested in interpreting reports of functions in other settings where consensus on basic issues such as confirmation of expression is lacking (Apps et al. 2008). The function of HLA-F is even more enigmatic with proposed roles in cross-presentation and NK receptor binding (Burian et al. 2016).

### MHC class II processing and presentation

MHC class II molecules represent a second group of surface receptors whose function is to present peptides to T cells, in this case various CD4 subsets. There are three classical isotypes, HLA-DP, HLA-DQ and HLA-DR, each composed of an alpha and a beta chain, which, with the exception of DR alpha, show extensive polymorphism. The exact number of alpha beta pairs expressed by an individual will vary depending on the number of alleles inherited from parents and their ability to pair in *trans*. MHC class II molecules show limited distribution and constitutive expression is largely limited to dendritic cells, B cells and cells of the monocyte/macrophage lineage but can be induced in other cell types (Unanue et al. 2016). During biosynthesis, alpha and beta chains assemble in the endoplasmic reticulum with a folding chaperone, invariant chain (Cresswell 1994). Targeting motifs encoded in the cytoplasmic tail of invariant chain direct the complex towards loading compartments termed MIIC (Neefjes 1999). During trafficking, Ii is sequentially degraded by proteases leaving a small peptide fragment, CLIP, in the binding groove. CLIP is exchanged for peptide that is generated by proteolysis of antigen that enters the endocytic pathway. Partial unfolding of protein is favoured by the reduction of inter or intra-molecular disulphide bonds by GILT, a thiol reductase and more generally by the low pH environment (Arunachalam et al. 2000). The protein is exposed to proteolysis, by a range of endopeptidases and exopeptidases, many of which show broad cleavage specificity (Lennon-Dumenil et al. 2002). Members of the cathepsin family are important not only for generating antigenic peptide but also for the degradation of invariant chain (Riese et al. 1996). Studies involving an asparginal endopeptidase suggest that key cleavage events may control subsequent generation of peptide epitopes and that proteolysis may not be as redundant as once thought (Antoniou et al. 2000).

Once generated, antigenic peptide can replace the surrogate peptide CLIP. This exchange process is facilitated by HLA-DM, a non-classical class II-related molecule (Kelly et al. 1991; Mellins and Stern 2014). Crystallographic studies have captured stages in this exchange process (Pos et al. 2012). Large conformational changes in DR alpha facilitate peptide dissociation and force incoming peptides to compete with repositioned DR residues for access to the P2 position and P1 pocket (Pos et al. 2012). Different alleles show varying degrees of DM dependency, suggesting pathways for DM-dependent and DM-independent CLIP exchange (Wieczorek et al. 2016). Additionally, polymorphic variants of DM show differential catalytic activity upon different class II substrates adding further complexity to the system (Alvaro-Benito et al. 2015). DM likely interacts with a natural transition state of class II, as it samples different conformations and is essential for efficient peptide loading by some alleles and less so for others (Wieczorek et al. 2016). As observed in computer-driven simulations, a natural plasticity in class II underpins both the DM-dependent and DM-independent exchange mechanisms (Wieczorek et al. 2016). Peptides loaded onto MHC class II in the presence and absence of HLA-DM may adopt different conformations that are recognised by non-overlapping T cell subsets (Mohan and Unanue 2012). The latter may escape thymic editing and predispose to autoimmunity (Mohan et al. 2010).

The activity of DM is controlled by DO, a molecule bearing close amino acid and structural similarity to DP, DQ and DR (Guce et al. 2013; Trowsdale and Kelly 1985). DO requires association with DM to prevent its degradation and to allow egress from the ER (Liljedahl et al. 1998). It adopts a conformation similar to HLA-DR when in association with DM, acting as a substrate mimic to block DM activity until it reaches a low pH environment (Guce et al. 2013). Under late endosomal/lysosomal conditions, acid-promoted destruction of DO occurs releasing free DM (Jiang et al. 2015). The role of HLA-DO is still controversial but likely involves fine-tuning of the peptide repertoire presented by MHC II, possibly by limiting the location of DM activity within the endocytic pathway or more specifically within the sub-domains of the multivesicular peptide loading compartment (Denzin and Cresswell 2013; Jiang et al. 2015; van Lith et al. 2001).

## Haplotypes and LD

The HLA region is under extensive linkage disequilibrium, leading to suggestions that the sets of polymorphic genes on haplotypes act in concert to coordinate presentation of the health of cells to the immune system. Just as it may be argued that by maintaining polymorphic TAP transporters near to class I genes allows for functional coordination on haplotypes, alleles of other genes within the MHC may be tuned as a set to balance immune responses. In relation to function of NK receptors, MHC haplotypes form two schools in which alleles are coordinated (Horowitz et al. 2016)). Other candidate genes for coordinated functional linkage include TNF, LTA, C2, C4, BF, MICA and MICB. This phenomenon may account at least in part for the extensive linkage disequilibrium over the region (Dawkins et al. 1999; Yunis et al. 2003). As new techniques for rapidly compiling complete MHC sequences replace laborious cloning and assembly more haplotypic relationships between genes will become evident (Horton et al. 2008; Lenz et al. 2015; Norman et al. 2016; Norman et al. 2015).

## Disease association

More diseases are associated with the MHC than with any other region of the genome (Lenz et al. 2016; Trowsdale and Knight 2013). The majority of these are autoimmune conditions. Two conditions where the MHC has been particularly informative are included in this volume, namely, Celiac disease (Sollid) and Arthritis (Kampstra and Toes). However, since the HLA region is so gene dense, polymorphic and spans 4Mbp, it is understandable that other disorders will be genetically associated. Included are several Mendelian disorders, associations of which are explained as linkage disequilibrium with HLA alleles. An example is congenital adrenal hyperplasia, which is due to alleles of the CYP21 genes, in the class III region (White et al. 1985). It is widely assumed that resistance to infection is driving the extreme MHC variation, although direct evidence for this is limited. Escape variants of HIV-1 are consistent with the need for continual novelty in peptide-binding grooves (Moore et al. 2002). Several viruses down-modulate HLA expression to escape T cell or NK cell recognition, which attests to its importance for disease resistance.

Genome-wide association studies (GWAS) for autoimmune conditions implicate contributions throughout the genome, but the MHC remains the most important link, with a greater effect size (Lenz et al. 2016). These disorders are generally associated with discrete class I or class II alleles. This is consistent with involvement of specific peptides. For example, narcolepsy, the sleeping disorder, is associated with HLA-DQB1*006:02 in at least two different populations (Mignot 1997). The recent finding that a specific flu vaccine gives rise to narcolepsy in a small group of young individuals is consistent with a complication of immune response to the vaccine (Ahmed et al. 2015). Narcolepsy is one of the best examples of a clear MHC association. It has been difficult to identify key MHC loci association in other disorders. This is likely because they may be influenced by more than one HLA locus. In addition, the gene density over the MHC and the strong LD make analysis very difficult, unless extremely large cohorts of patients and controls are studied to generate sufficient statistical power. For example, sarcoidosis is an autoimmune disease associated with HLA-DRB1*003. There are reports of an additional, independent association with BTNL2, but these are difficult to confirm, since the two genes DRB1 and BTNL2 are adjacent (Valentonyte et al. 2005). Most autoimmune conditions are multifactorial and appear to involve many gene variants as well as environmental effects. Clearly, more reliable data may be obtained by larger studies. This has been facilitated recently by imputation of HLA type to 4-digit resolution based on high density SNP genotyping (Leslie et al. 2008). Infectious diseases associated with the MHCs of a variety of species are covered in detail in this volume.

There has been progress in understanding one set of MHC-associated conditions, namely drug sensitivities (Illing et al. 2012). This topic is reviewed here by the key contributors to understanding the molecular mechanisms behind the pharmacogenomics; McClusky this volume. Abacavir sensitivity is strongly associated with HLA-B*057:01. Purification of HLA-B*057:01 from cells treated with abacavir indicated that the drug bound to the antigen-binding cleft, pushing up peptides and thereby leading to stimulation of a set of T cells. The drug non-covalently bound to the B*057:01 molecule, breaking tolerance (Illing et al. 2012).

Other conditions linked to the MHC region include cancers of a suspected viral aetiology, such as nasopharyngeal carcinoma and Hodgkin’s lymphoma. There are reports of a variety of other condition such as schizophrenia and complement C4 genes, embedded in the MHC (Sekar et al. 2016).

## Receptors

As discussed above, antigen presentation is probably the main raison d’etre of class I and class II molecules. Both classes I and II interact with T cells, through their receptors, which result in modulation of immune responses in a peptide-dependent fashion. But, there are a variety of ways in which the information from these molecules may be interpreted. Other sets of receptors may detect differences in MHC classes I and II in peptide-dependent and peptide-independent ways resulting in ‘missing-self’, releasing inhibition by inhibitory receptors on NK or other cells, as discussed in this issue.

KIRs bind sets of HLA molecules generally assumed to be associated with peptide (Saunders et al. 2015). Changes in the peptide associated with class I molecules can prevent inhibition of NK activation (Fadda et al. 2010). In this regard, they have peptide:HLA specificity, which may be associated with effects on infections with viruses. Examples include the inhibitory receptor KIR2DL3 and hepatitis C and the activating receptor KIR3DS1 and HIV (Buchanan et al. 2015; Martin and Carrington 2013). The main paradigm for Natural Killer cells detecting the presence of class I molecules is encapsulated in the missing self hypothesis, whereby loss of class I or failure to bind to inhibitory receptors signifies that the cell has been compromised. Canaries were taken down coalmines as a warning system for toxic gasses, particularly carbon monoxide. In a similar way, class I molecules are sentinels of unhealthy or infected cells. Some KIR-related receptors on cells of monocyte lineages (LILR, ILR, ILT) also influence immune responses.

## Cancer immunosurveillance

There is some evidence that the immune system naturally protects against cancer (Corthay 2014). However, the incidence of cancer only increases marginally when acquired immunity is disabled, and it has also been proposed that inflammation may promote tumour growth (Mantovani et al. 2008).

In spite of this, initiatives to direct adaptive T cell immunity to cancer cells are starting to show promise. There has been some clinical success with three approaches: (a) cancer vaccination; (b) with antibodies that stimulate T cells by blocking inhibitory receptor interactions, such as anti-CTLA4, PD1 or PDL1; and (c) with adoptive transfer of anti-tumour T cells. These techniques depend on exploiting T cells from the patients that specifically recognise tumour antigens through MHC molecules. They may be combined to produce greater efficacy.

Before discussing issues associated with MHC and immunosurveillance, it is worth considering the scope of HLA expression. The textbook view is that class I is expressed on all nucleated cells, with a few notable exceptions, and that class II molecules are restricted in expression to professional antigen presenting cells unless induced by cytokines such as gamma-interferon. This simplistic view has been questioned as there is evidence for considerable variation in HLA levels in different tissues. There is debate over whether loss of HLA expression in tumours is governed by immunoselection or whether it is simply a by-product of genomic instability (Campoli et al. 2012).

There are at least three major problems in terms of specificity:

1. Loss of HLA class I or peptide epitope expression—immunoediting

There is abundant evidence for loss of expression of HLA class I as an escape mechanism on a variety of tumours (Garrido et al. 2016). A number of strategies have been suggested to overcome HLA class I defects if they are due to reversible mechanisms and not permanent gene deletion (Lampen and van Hall 2011). These include addition of cytokines or the use of epigenetic modifiers such as inhibitors of DNA methyl transferase or histone deacetylase. However, ~30–40% of tumours appear to have a more permanent loss of HLA class I, due for example to loss of β2microglobulin. In principle, NK cells may be capable of targeting HLA-negative tumours, if they are stimulated by IL-12/IL-18 treatment. It has been proposed that tumours with a more complex genome have a higher frequency of total HLA class I loss (Rashidi 2014). There are also data suggesting that there may not be a simple relationship between class I and cancer survival (Powell et al. 2012).

2. Identification of epitopes

After some early work identifying mouse tumour antigens, pioneering work from Thierry Boon’s laboratory revealed novel epitopes in human cancers (Coulie et al. 2014). The first of these, derived from the MAGEA1 gene, was a normal cellular component from a gene family. Other anti-tumour cytotoxic T cells (CTLs) were shown to recognise peptides from antigens that were mutated in the tumour cells. In melanoma, some CTLs and some CD4 T cells recognised peptides from proteins specifically expressed in the melanoma cells. It was realised that numerous genes are likely to be mutated in cancer, producing neo-epitopes that could be presented by the specific MHC allele in the patient. Computer algorithms were generated to identify likely peptide sequences that could be presented by the host’s MHC molecules. In essence, there are four types of tumour antigen recognised by T cells: (a) viral antigens, in cervical carcinoma or hepato-carcinoma, where there is a clear viral aetiology; (b) antigens from mutated genes, by a variety of mechanisms, including frame-shift, single amino acid changes or protein extension due to loss of the correct stop codon; (c) cancer-germline genes, that also used to be called oncofetal antigens. These are normal proteins expressed in tumours but not in normal somatic tissues. They may have become activated in tumours due to demethylation of their promoter. (d) Proteins over-expressed in tumours. An example of this is MUC1, a protein over-expressed on most adenocarcinomas.

Recent work indicates that peptide targets for CTLs are not randomly distributed amongst the whole range of cellular proteins but rather are focussed onto less than 50% of genes, from selective regions of the genome (Pearson et al. 2016). Some proteins are responsible for several different peptide antigens, and peptides may be derived from non-canonical reading frames (Laumont et al. 2016). Algorithms are being developed to facilitate prediction of relevant self peptides. Another complication is the finding that a large number of HLA-associated peptides are ‘spliced’ products of the proteasome (Liepe et al. 2016). There are data that suggest tumours that respond best to immunosurveillance have the highest mutagenic load (Alexandrov et al. 2013).

3. Autoimmune side effects

It is difficult to predict whether T cell immunotherapy to a specific antigen will be harmful, but several cases have been documented (Coulie et al. 2014). Most immunotherapeutic approaches to cancer have concentrated on T cells, but there is emerging interest in NK cells in the last few years (Childs and Carlsten 2015). Clearly, there is a long way to go in developing better tumour immunotherapy. There is evidence that the local tumour environment is immunosuppressive. It is possible that therapeutic intervention invokes immunostimulation, awakening quiescent T cells already in the tumour environment (Coulie et al. 2014). Introducing inhibitors of immunosuppression may help to complement some of the approaches.

Contributions to the subject of HLA and cancer are included in this volume.

Peptide databases: iedb (http://www.iedb.org/); SYFPEITHI (http://www.syfpeithi.de/); http://www.imtech.res.in/raghava/mhcbn/.
